# Fracture Related Infections and Their Risk Factors for Treatment Failure—A Major Trauma Centre Perspective

**DOI:** 10.3390/diagnostics12051289

**Published:** 2022-05-22

**Authors:** Victor Lu, James Zhang, Ravi Patel, Andrew Kailin Zhou, Azeem Thahir, Matija Krkovic

**Affiliations:** 1 School of Clinical Medicine, University of Cambridge, Cambridge CB2 0SP, UK; zz357@cam.ac.uk (J.Z.); azhou1998@icloud.com (A.K.Z.); 2 Hull York Medical School, University Rd, Heslington, York YO10 5DD, UK; hyrp16@hyms.ac.uk; 3 Addenbrookes Hospital, Hills Rd, Cambridge CB2 0QQ, UK; azeem.thahir@addenbrookes.nhs.uk (A.T.); matija.krkovic@addenbrookes.nhs.uk (M.K.)

**Keywords:** fracture, infection, trauma, amputation, polymicrobial infection, risk factor

## Abstract

Fracture related infections (FRI) are debilitating and costly complications of musculoskeletal trauma surgery that can result in permanent functional loss or amputation. Surgical treatment can be unsuccessful, and it is necessary to determine the predictive variables associated with FRI treatment failure, allowing one to optimise them prior to treatment and identify patients at higher risk. The clinical database at a major trauma centre was retrospectively reviewed between January 2015 and January 2021. FRI treatment failure was defined by infection recurrence or amputation. A univariable logistic regression analysis was performed, followed by a multivariable regression analysis for significant outcomes between groups on univariable analysis, to determine risk factors for treatment failure. In total, 102 patients were identified with a FRI (35 open, 67 closed fractures). FRI treatment failure occurred in 24 patients (23.5%). Risk factors determined by our multivariate logistic regression model were obesity (OR 2.522; 95% CI, 0.259–4.816; *p* = 0.006), Gustilo Anderson type 3c (OR 4.683; 95% CI, 2.037–9.784; *p* = 0.004), and implant retention (OR 2.818; 95% CI, 1.588–7.928; *p* = 0.041). Given that FRI treatment in 24 patients (23.5%) ended up in failure, future management need to take into account the predictive variables analysed in this study, redirect efforts to improve management and incorporate adjuvant technologies for patients at higher risk of failure, and implement a multidisciplinary team approach to optimise risk factors such as diabetes and obesity.

## 1. Introduction

Fracture fixation and management is a difficult task due to the unpredictable and sometimes unstable nature of bone damage. This is further complicated by the presence of concomitant injuries and comorbidities, and the numerous perioperative and postoperative complications that could arise after emergency treatment. Fracture-related infection (FRI) is a serious and challenging complication in musculoskeletal trauma, which could result in delayed healing, permanent functional loss, or amputation, leaving a long-lasting and catastrophic impact on a patient’s quality of life. Epidemiological studies suggest an infection rate of 1.8% following closed fractures and 27% following open fractures [1]. FRI cases generate 6.5 times higher healthcare costs than non-infected cases, with reported treatment success rate at 70%, a recurrence rate of 9% and amputation rate of 3% [2].

Multiple factors can make the management of FRIs challenging, such as open fractures, soft tissue contamination, perioperative illness, polytrauma, multiple surgical procedures, and exposure to nosocomial bacteria during prolonged hospital stays [3]. Open reduction internal fixation (ORIF) is a common method for fracture fixation, requiring internal hardware to remain for a period of time to allow for fracture stabilisation and union. This however creates an ideal environment for infection [4]. Soft tissue damage and vascular compromise encourage microbial infiltration which upsets normal bone healing [3]. Furthermore, a positive feedback mechanism leads to a vicious cycle of biomechanical instability at the fracture site and FRI, as described by Perren’s concept of strain theory and fracture healing [5,6]. Fracture instability leads to impaired neovascularistaion; together with ongoing osteolysis and soft tissue damage, this encourages microbial proliferation and undermines the host immune response. This leads to further biomechanical instability [7].

The basic paradigm for FRI management involves irrigation, debridement, soft tissue management, osseous stability, identifying involved microbes, and targeted antibiotic therapy. This has been recently supplemented by a more extensive paraphernalia for FRI management, such as Masquelet technique, reamer irrigator aspirator, and antibiotic cement rods [8]. Although there seems to be a decrease in FRI incidence and increase in FRI treatment success over the previous decade [9], it is prudent to ask if we have arrived at a plateau using current technology and guidelines. Whilst the rates and predictive variables for other musculoskeletal conditions such as periprosthetic joint infections (PJI) and septic arthritis are well described [10,11,12], the literature surrounding the risk factors of FRI are comparatively scarce. The aim of this study was to identify risk factors for FRI management failure at a major trauma centre over a six-year period, allowing for the development of a prognostic algorithm in clinical practice.

## 2. Materials and Methods

### 2.1. Study Design

After approval by the institutional review board on 18 March 2021 (registration number 9670), a retrospective observational study was performed at our major trauma centre from January 2015 to January 2021. Patients met inclusion if they were over the age of 18 and underwent a surgical procedure for an open or closed fracture, following which surgical irrigation and debridement (I&D) was performed due to clinical, microbiological, or radiological signs of infection. Infections that do not communicate with the fracture or implant such as pin site infections and cellulitis, patients with PJIs of the hip and knee joint, and those without prior fracture fixation were excluded. Soft tissue infections, diabetic foot ulcers, pressure sore infections, patients transferred to other institutions, and patients lost to follow-up within 12 months post-injury were excluded. All clinical records and radiographs were reviewed by two authors (VL and JZ) independently, who were not involved in patient treatment process, thus avoiding assessment bias.

Open fractures were managed according to BOAST guidelines [13], and were subdivided according to the Gustilo-Anderson (GA) classification [14], as determined during debridement in the operating theatre. Polytrauma was classified when the Injury Severity Score (ISS) was 16 or greater [15]. An infection was defined as purulent discharge, erythema, and/or surgical wound dehiscence exposing underlying hardware following definitive fixation and wound closure, necessitating a return to the operating theatre for I&D. At least two separate deep tissue/implant specimens taken during the surgical I&D, returning phenotypically indistinguishable pathogens must be collected, following the consensus statement from an international expert group [16]. Infections were stratified into acute and chronic, which represent infection onset less than or more than six weeks post-injury, respectively. Infections were not subdivided into superficial or deep, following an agreement from an international expert group [17]. The superficial nature of a FRI can only been determined retrospectively and should not be used to guide treatment [17].

FRI treatment failure was defined by infection recurrence or amputation. Infection recurrence was defined as the unscheduled return to operating theatre for further debridement or implant revision due to new clinical indications of infection, followed by a new course of antibiotic therapy.

### 2.2. Treatment Protocol

**Figure 1:**
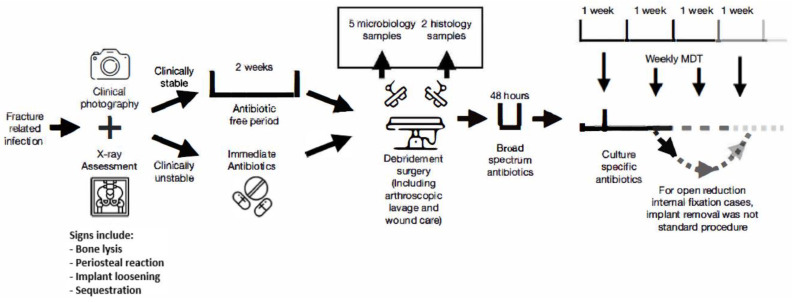
Treatment protocol.

Figure 1 describes our FRI treatment protocol according to British Orthopaedic Association FRI guidelines [18]. For suspected FRI cases, plain radiology and CT scans with contrast were performed to assess features of osteomyelitis such as bone lysis, periosteal reaction, implant loosening, or sequestration, and clinical photography of wounds were taken, with images kept in patients’ files for comparison at subsequent multidisciplinary team (MDT) meetings. To facilitate FRI assessment, debridement surgery was performed, comprising arthroscopic lavage, wound care, five samples around fracture site for microbiological culture using separate instruments, and two samples for histological sampling. For stable patients, a two-week antibiotic-free period was kept before sampling, whilst patients with signs of systemic sepsis were given parenteral antibiotics immediately.

A bone infection specialist prescribed broad-spectrum antibiotics after sampling, and this was made culture-specific within 48 h following preliminary culture results. Empiric antibiotic therapy was given to patients with culture-negative results. FRI patients were the subject of weekly bone and joint infection MDT review to monitor antimicrobial management and surgical strategy. For those undergoing ORIF, implant removal was not planned as a standard procedure. The way implants were handled (removed or retained) following the initial FRI diagnosis was documented.

### 2.3. Statistical Analysis

Statistical analysis was performed using IBM SPSS Statistics version 27. Categorical variables were analysed with Fischer’s exact test (*n* < 10) or Chi-squared test (*n* ≥ 10); nonparametric continuous variables were analysed using Mann–Whitney U test. Data was also examined using univariable logistic regression models for the primary outcome measure FRI management failure and sixteen prognostic variables. Although some studies have advocated the use of a ‘ten events per variable’ criterion for logistic regression analyses, large scale simulation studies have found weak evidence supporting this criterion [19]. Co-variates with a *p*-value *p* < 0.100 in the univariable analysis were included in a multivariable logistic regression model, which then underwent a backwards elimination process to identify independent associations with FRI treatment failure. Statistical significance was defined as *p* ≤ 0.05.

## 3. Results

**Figure 2:**
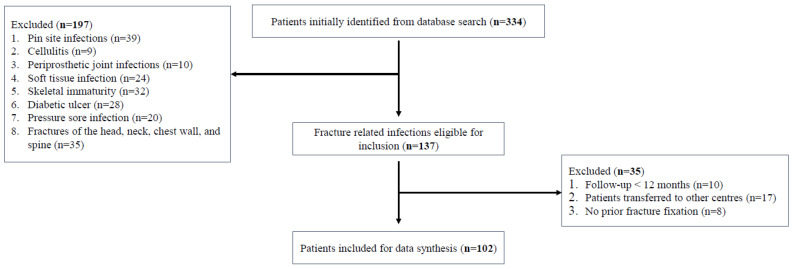
Flow diagram showing number of patients at each stage of the study.

During the six-year study period, 334 patients were identified at from our electronic hospital records, with 102 FRIs in 102 patients being included (Figure 2). With a total of 8831 fractures treated over the study period, this represents an overall 1.16% infection rate. Table 1 shows patient demographics and Table 2 shows the location of FRI. In total, 66 males and 36 females were included, with 35 open fractures, and 67 closed fractures. The mean age at injury was 49.71 years old. Fall from height was the main cause of injury (34.3%). In all, 78 (76.5%) of patients had purulent discharge, 76 (74.5%) had erythema, and 58 patients (56.9%) had wound dehiscence (Table 3). Moreover, 56 patients (50%) had all three observations, the remaining patients had at least two separate deep tissue/implant specimens returning phenotypically indistinguishable pathogens. Regarding cardiovascular risk factors, 37 patients were current smokers (36.3%), 31 patients were diabetics (30.4%), and 32 patients (31.4%) were obese (BMI ≥ 30.0).

There were 35 open fractures and 67 closed fractures. As such, 87 patients were treated with ORIF, amongst which sixty received an intramedullary nail. Fluoroquinolones were the most common culture-specific antibiotic prescribed (Table 4). The most common culprit bacteria belonged to the *Staphylococcus* family. Sixteen patients (15.7%) had signs of systemic sepsis, and received immediate blood cultures and parenteral antibiotics. Total follow-up time was on average 2.37 years (range: 12.46–61.67 months). Ten patients who were lost to follow-up within 12 months post-injury were excluded.

Of the 102 patients, 30 (29.4%) had an acute infection (onset < 6 weeks). FRI treatment failure occurred in 24 patients (23.5%). Twenty-one (20.6%) had recurrent infection and three (2.9%) required an amputation (Table 5). Fourteen of those were polytrauma patients. Univariable logistic regression model showed that obesity (BMI ≥ 30) (*p* = 0.025), GA type 2 (*p* = 0.080, GA type 3a (*p* = 0.091), GA type 3b (*p* = 0.031), GA type 3c (*p* = 0.033), polymicrobial infection (*p* = 0.011), and implant retention (*p* = 0.048) were associated with FRI treatment failure (Table 6). Multivariable logistic regression model showed the risk factors associated with FRI management failure were obesity (OR, 2.522; 95% CI, 0.259–4.816; *p* = 0.006), GA type 3c (OR, 4.683; 95% CI, 2.037–9.784; *p* = 0.004), and implant retention (OR, 2.818; 95% CI, 1.588–7.928; *p* = 0.041).

## 4. Discussion

Despite any rigorous prevention strategy against FRIs, this challenging complication still frequently occurs, with failure of initial treatment resulting in further debridement and recurrent infections, leading to increased morbidity and socio-economic burden. The FRI treatment failure rate of 23.5% is at the lower end of that reported in the literature [20], but is still unacceptably high. This study found that obesity, implant retention, and GA type 3 open fracture are risk factors for FRI treatment failure. GA type 3 was also reported as a risk factor by Horton et al. [21]. These results however differ with a recently published study, who found that repeat I&D procedures and culture-negative infections (CNIs) were risk factors for FRI treatment failure [20]. Gitajn et al. also underlined the importance of CNIs [22], however both studies had relatively low numbers of CNIs, and literature surrounding PJIs suggest that CNIs have equal or better outcomes than their culture-positive counterparts [23,24,25]. Similarly, the association of multiple I&Ds with treatment failure was also suggested by other studies [26,27]. However, this is likely biased by patients with more severe infections needing multiple I&Ds, as well as the surgical protocol utilised, namely a repeat operative I&D every 2–3 days [20]. Our protocol utilised a more intensive and thorough debridement, rather than repeated surgical insult which increases bacterial colonisation risk and prolongs fracture healing [28].

### 4.1. Polymicrobial Infections

Polymicrobial infections, whilst not reaching statistical significance on the multivariable model, increased treatment failure risk in the univariable model. Difficult to treat organisms such as *Enterococcus* and Gram-negative bacilli were suggested to be the most common culprits in polymicrobial infections [29]. They require complex antibiotic coverage, and prolong treatment duration, with one study on prosthetic joint infections recording a 0.3 OR between polymicrobial infection and being free of infection after two years [30]. Studies have suggested that this may be due to microbial synergy that enhances virulence and decreases susceptibility to host immune defences, caused by mechanisms such as metabolite cross-feeding [31], quorum sensing [32], and synergistic biofilm formation [33].

The most common isolates were *Staphylococcus* and the Gram-negative bacilli *Pseudomonas*. No multidrug resistant (MDR) *Pseudomonas* was recorded, despite a 14% chance of becoming so [34], but encountered four cases of extended-spectrum beta-lactamase-producing *Pseudomonas* which limited choices for antibiotic therapy. Nevertheless, in clinical practice, drug allergies are often the limiting factor for antibiotic therapy choices; twelve patients (11.8%) were allergic to penicillin or cephalosporins. We noticed that polymicrobial infections diagnosed at first culture were more likely to lead to treatment success than those identified during subsequent cultures (14/20, 70% vs. 4/10, 40%; *p* = 0.114), agreeing with Kavolus et al. [35]. Furthermore, obese patients had higher rates of polymicrobial infection than non-obese patients (14/32, 43.8% vs. 16/70, 22.9%; *p* = 0.032), agreeing with a cohort study of PJIs [36].

### 4.2. Obesity

Obesity was a risk factor for FRI treatment failure on both univariable and multivariable models. Obesity influences the risk of either acquiring an infection or outcome of an infection [37], with a meta-analysis showing obesity increasing surgical site infection risk by two-fold [38]. This could be due to high levels of subcutaneous fat, increasing surface tension at the surgical site, prolonged wound drainage [39], increased bacterial skin colonisation [40], or impaired cross-talk between adipocytes, adipokines, and leukocytes, leading to dysregulation of immune system and altered macrophage differentiation [41]. One cohort study reported that compared to the non-obese, obese patients had increased reoperation for infectious complications (13.3% vs. 34.2%; *p* = 0.014), and a higher cumulative incidence of sepsis (9.52 vs. 15.85 events/100 patient-months) [42].

Obesity affects pharmacokinetics, by increasing volume of distribution (V_d_) of lipophilic drugs such as macrolides and fluoroquinolones, and decreasing V_d_ of hydrophilic drugs such as aminoglycosides. This leads to frequent underdosing of antimicrobials in obesity [43], with a multicentre study showing insufficient plasma concentrations of vancomycin in obese patients when standard doses were used [44]. Additionally, in vivo studies in mice suggested that obesity leads to impaired bone healing and non-union, by reducing levels of key proteins responsible for recruitment of mesenchymal stem cells and their differentiation into osteoblasts [45], or by increasing callus adiposity leading to biomechanical weakness [46].

### 4.3. Diabetes

Diabetes increased treatment failure risk in the univariable model but did not reach statistical significance on the multivariate model. Diabetes is a risk factor for acquiring infections, with one retrospective cohort study involving more than 1,000,000 participants reporting a risk ratio for infectious disease-related hospitalisation of 2.17 (99% CI 2.10–2.23) for diabetic vs age-matched controls, and a risk ratio for infection-attributed death of 1.92 (99% CI 1.79–2.05) [47]. Studies have suggested that diabetes leads to immune dysfunction, by crippling the innate immune system by reducing phagocytic activity of macrophages and neutrophils, and impairing activation and performance of the adaptive immune system, hence complicating the course of infection in diabetics [48].

A meta-analysis of 1637 subjects demonstrated that diabetics had higher odds of antimicrobial resistant infections (OR = 2.35; 95% CI, 1.49–3.69) [49]. This could be due to diabetics visiting hospitals more often for diabetes clinics or other complications, during which they may acquire nosocomial infections, which are more likely to be resistant [50]. Additionally, diabetes can influence antibiotic effectiveness by altering gut microbiota [51], slow gastric emptying [52], or via diabetic medication and antibiotics pharmacological interactions. A longitudinal cohort study of 92 participants with *S. stercoralis* infection showed that diabetes is significantly associated with treatment failure [53]. Furthermore, diabetes negatively influences fracture healing by compromising osteoblastogenesis [54], and producing advanced glycation end products which decreases bone material strength [55]. A retrospective study of 190 fractured calcanei reported a relative risk for non-union of 3.4 (*p* = 0.02) for diabetics vs. age-matched controls [56].

### 4.4. Implant Retention

For definitive fixation, 87 patients received ORIF, sixty of whom received an intramedullary nail. Implant retention was associated with a higher risk of FRI treatment failure on both univariable and multivariable models. Metal implants promote biofilm colonisation, hence acting as a focal point for bacterial colonisation [57]. The literature is sparse and dived regarding the dilemma of implant retention or removal during treatment of an infection following a fracture manged by ORIF. A retrospective study of patients with an infection less than sixteen weeks after ORIF found a higher infection recurrence rate in those treated with hardware retained than removed (36% vs. 16%) [58]. Nevertheless, some studies report no effect of implant removal or retention on FRI treatment efficacy [59], whilst some report the opposite, with implant removal being a significant risk factor for FRI treatment failure [20,21]. This could be due to selection bias, since surgeons could have removed more implants in patients who had more severe infections.

Especially in the early stages, implant retention is commonplace since removal would disturb fracture healing, leading to non-union. Berkes et al. performed a similar study to Rightmire et al. but used a cut-off point of six weeks for acute infections rather than sixteen weeks [60] when bony union is tenuous. Berkes et al. found that 71% of patients with hardware retention achieved fracture union. The conclusion was that implants can be maintained if sufficient debridement of implant site with appropriate antibiotic therapy are carried out, and is only promising in acute/early-onset FRIs occurring prior to bony union [60]. This was confirmed by another study investigating debridement, antimicrobial therapy, and implant retention (DAIR) in FRIs, whereby chronic/late-onset FRIs treated with DAIR were associated with higher infection recurrence rate [61]. This agrees with our cohort, whereby those with implant retention were more likely to have successful FRI treatment if they had an acute infection (<6 weeks) than a chronic infection (*p* = 0.146; OR = 2.647; 95% CI, 0.696–10.065). The balance between maintaining fracture stability for osseous union by retaining the implant vs. the concern for biofilm growth is another challenging aspect of FRI management.

### 4.5. Gustilo-Anderson (GA)

Severity of open fractures according to GA classification, especially GA type 3c, had unexpectedly the highest odds for FRI treatment failure on both univariable and multivariable models. Only one other study published results similar to ours [21]. GA type 3 injuries are greatly contaminated and require vascular and flap reconstruction due to neurovascular damage and extensive soft tissue injury, which makes it harder to clear infections. Extensive wound contamination results in a greater risk of polymicrobial infection [62], which is itself a risk factor for FRI treatment failure. Furthermore, open fractures with a high GA classification are associated with lengthier stays in hospitals [63], which may lead to increased chance of acquiring nosocomial infections [50].

For severe open fractures, studies have shown that bone debridement with flap coverage was associated with better osteomyelitis outcome, by improving perfusion and coverage [64]. A cohort study of 34 patients suggested that a muscle flap provides effective treatment for chronic osteomyelitis wounds [65], however the selection of a pedicled, muscle, fasciocutaneous, or free flap should be made according to the defect characteristics.

### 4.6. FRI Prevention and Characteristics

Given the dire socio-economic consequences of FRIs, recent guidelines have reiterated the need for FRI prevention, such as soft tissue coverage preferably within 72 h of injury, and no later than 7 days [66]. With a range of 14–80 h, all included patients met this criteria. Furthermore, studies suggested that a one week delay in wound coverage increases the risk for recurrent infection [67], however those who suffered recurrent infections did not have a longer time to soft tissue coverage. Guidelines also suggest that discharge documentation should make clear to primary carers and patients on how to respond in the event of a suspected FRI [18], yet this was only seen in two of the included patients (1.96%). This is not a statistic reported in the literature, but warrants greater attention from clinicians.

Willenegger and Roth classified FRIs according to time of onset into early (<2 weeks), delayed (2–10 weeks), and late-onset (>10 weeks) [68]. Despite being used to guide treatment in some studies, the frequency of each subtype is often not quoted in studies, with other authors electing to subdivide infections into acute (<6 weeks), and chronic (>6 weeks) [69]. It can be argued that these time frames are arbitrary and not rooted in scientific evidence. Furthermore, the definitions are heterogenous, with some defining time since injury or time since symptom onset. A recent systematic review found a large variety of time windows used to classify infections [2], with 71% of included studies failing to report time in their classification of infection.

Nevertheless, we stratified acute and chronic infections based on infection onset occurring less or more than 6 weeks after injury, respectively. This was performed to reflect the fact that treatment choices should differ, since for early infections within the first two weeks, the bone shows no sign of osteolysis or osteomyelitis, despite the presence of bacteria [17]. Hard callus formation occurs after 3 weeks, during which biofilms mature and become more resistant to antibiotic therapy, and bacterial bone invasion occurs [70]. Indeed, 75.0% (12/16) of bone defects after radical debridement and 61.2% (30/49) of implant removal occurred in those with chronic infections, and those with an acute infection needed on average a shorter antibiotic treatment duration than those with a chronic infection.

The bacteria family responsible for the most infections was *Staphylococcus*, agreeing with prior studies [20]. *Staphylococcus aureus* was responsible for eleven FRIs, among which seven occurred in those with acute infections. This could be because *Staphylococcus aureus* is a more virulent bacteria, which is predominantly acquired as a direct consequence of trauma [70]. As per the results of two systematic reviews [2,70], it is commonly accepted that purulent discharge and surgical wound dehiscence (SWD) are pathognomonic of FRIs. However, the majority of the included patients did not show wound dehiscence (54/102; 52.9%). Some clinicians view SWD as synonymous with FRI, however rates of infection in dehisced wounds are infrequently reported [71], and is often associated with other factors such as diabetes [72]. Eighteen diabetics in our cohort (18/31; 58.1%) had wound dehiscence listed as a finding. Furthermore, in studies that do report infection rates, it is unclear if infection occurred before or after SWD. Although classified as ‘suggestive criteria’ [73], the vast majority of the included patients reported pain (96/102; 94.1%), redness (76/102; 74.5%), and swelling (77/102; 75.5%), and hence should be also given a high level of importance when assessing FRIs. Laboratory findings (ESR, CRP, WBC) were recorded but studies have questioned their usefulness in diagnosing FRIs, with the area under the receiver operating characteristic (AUROC) of the combined markers being 0.63 [74]. A meta-analysis has also reported poor specificity and sensitivity [75]. All patients had X-ray and CT scans with contrast to look for radiological signs of FRI, but only 24 (23.5%) had an MRI scan. Plain X-ray scans have poor sensitivity for detecting FRI, especially in the early stages, with a much improved result for CT or MRI scans [76]. Nuclear imaging techniques have recently been developed to improve the diagnostic accuracy such as FDG-PET and WBC scintigraphy. Despite good evidence that FDG-PET has high sensitivity and specificity, there is a lack of studies focusing on the use of WBC scintigraphy for FRI [77].

### 4.7. Limitations

Being a retrospective observational study, the accuracy of patient’s notes and attendance at follow-up clinics were relied upon. The patients were treated at a major trauma centre with a dedicated bone infection team, thus patients were unlikely to be followed up elsewhere for their FRI management. There was a lack of a standardised protocol for diagnosis of infection, which relied on the clinical assessment of the consultant in-charge, but is consistent with many prior studies [17,20,21]. Nevertheless, the use of consensus recommendations on diagnosis added strength to thus study [16]. Implant type was not analysed, with studies suggesting an association between implant surface area and biofilm formation [17]. Finally, nearly half the included patients (46.1%) sustained major trauma according to the Injury Severity Score, who are more likely to have a poorer prognosis and develop a FRI [78]. Nevertheless, this retrospective observational study studied a wide-range of patient factors that could play a role in FRI treatment failure, involved a six-year time span of patients treated at a major trauma centre, enhancing the external validity of our results. This study is well suited to analyse risk factors for FRI treatment failure given the long overall follow-up time of 2.37 years, and mean follow-up time of 1.78 years after initial FRI treatment ended, allowing us to catch the majority of treatment failures.

## 5. Conclusions

One of the most challenging complications in musculoskeletal surgery is a FRI. Lowering the burden requires a three-pronged approach: a uniform set of standards for the prevention of infections in trauma and elective orthopaedic surgery, a robust diagnostic workup for any suspected FRI case, and an effective management scheme for confirmed FRI cases that differentiates between chronic and acute infections given their different pathophysiologies. 

We identified obesity, open fractures with high Gustilo-Anderson grade requiring flap and vascular reconstruction, and implant retention as predictive factors for FRI treatment failure. Successful management includes, developing alternative treatment strategies and incorporating adjuvant technologies for higher risk patients such as next generation sequencing of bacterial samples, and optimising modifiable risk factors before treatment such as diabetes and obesity. The trauma literature could be supplemented with primary studies with large cohorts and adequate follow-up time, whereby risk factors were identified and mitigated, with this study potentially contributing to the development of a standardised protocol.

## Figures and Tables

**Table 1:** Patient demographics.

Total Population	102
Male	66 (64.7%)
Female	36 (35.3%)
Mean age at injury in years (range)	49.71 (18–87)
Male	48.25 (18–84)
Female	52.38 (22–87)
BMI (range)	27.08 (16.12–47.70)
Open fracture	35 (34.3%)
Gustilo-Anderson 2	6
Gustilo-Anderson 3a	13
Gustilo-Anderson 3b	11
Gustilo-Anderson 3c	5
Closed fracture	67 (65.7%)
Smoking status
Ex-smoker	33 (32.4%)
Non-smoker	24 (23.5%)
Current smoker	45 (44.1%)
Diabetes Mellitus
Yes	31 (30.4%)
No	71 (69.6%)
Fracture Mechanism
RTC (high energy)	33 (32.4%)
Fall from height (high energy)	35 (34.3%)
Trip and fall (low energy)	27 (26.5%)
Crush trauma (low energy)	6 (5.9%)
Gunshot wound (high energy)	1 (1.0%)
Polytrauma (ISS ≥ 16)
Yes	49 (48.0%)
No	55 (52.0%)
Definitive Fixation
Open reduction internal fixation (ORIF)	87 (85.3%)
External Fixation	15 (14.7%)
Reamed *
Yes	53 (88.3%)
No	7 (11.7%)

* Intramedullary nail only.

**Table 2:** Location of fracture related infection.

Humerus Shaft	5 (4.9%)
Radius	8 (7.8%)
Olecranon/Ulna	9 (8.8%)
Hip	9 (8.8%)
Neck of femur	6 (5.9%)
Femoral shaft	17 (16.7%)
Tibial plateau	5 (4.9%)
Tibial shaft	30 (29.4%)
Ankle	12 (11.8%)
Clavicle	1 (1.0%)

**Table 3:** Parameters of infection present in FRI patients.

**Clinical Signs**	
Fever	7 (6.9%)
Purulent discharge	78 (76.5%)
Wound dehiscence	58 (56.9%)
Dolor (pain)	96 (94.1%)
Rubor (erythema)	76 (74.5%)
Tumor (swelling)	77 (75.5%)
**Radiological Signs**
Osteomyelitis signs ^†^	81 (79.4%)
Evidence of non-union	19 (18.6%)
**Bacteriological Testing**
Ultrasound-guided aspiration	22 (21.6%)
7 cultures taken *	71 (69.6%)

* BOA FRI guidelines states that during debridement surgery, 5 samples must be taken for microbiological culture, and 2 samples for histology. ^†^ These include periosteal bone formation, bone lysis at the fracture site or around the implant, implant loosening, or sequestration.

**Table 4:** Antibiotics and Microbes.

Initial Culture-Specific Antibiotic		
Penicillin	14 (13.7%)	
Cephalosporin	13 (12.7%)	
Tetracycline	7 (6.9%)	
Aminoglycoside	4 (3.9%)	
Macrolide	2 (2.0%)	
Fluoroquinolone	18 (17.6%)	
Glycopeptide	15 (14.7%)	
Carbapenem	14 (13.7%)	
Oxazolidinone	3 (2.9%)	
Lipopeptide	9 (8.8%)	
Lincosamide	3 (2.9%)	
Culprit Bacteria Family		
	Polymicrobial (*n* = 34)	Monomicrobial (*n* = 63) *
Aeromonas	1 (2.9%)	1 (1.6%)
Enterobacter	10 (29.4%)	10 (15.9%)
Pseudomonas	29 (85.3%)	10 (15.9%)
Enterococcus	21 (61.8%)	11 (17.5%)
Staphylococcus	23 (67.6%)	12 (19.0%)
Salmonella	0 (0%)	2 (3.2%)
Cutibacterium	5 (14.7%)	2 (3.2%)
Proteus	2 (5.9%)	2 (3.2%)
Streptococcus	9 (26.5%)	6 (9.5%)
Corynebacterium	1 (2.9%)	3 (4.8%)
Others	6 (17.6%)	4 (6.3%)
One infectious organism	N/A	63 (100%)
Two infectious organisms	7 (20.6%)	N/A
Three infectious organisms	15 (44.1%)	N/A
Four infectious organisms	12 (35.3%)	N/A

* Five patients had culture-negative infection.

**Table 5:** Injury and FRI characteristics.

	Total (*n* = 102)	Open Fractures (*n* = 35)	Closed Fractures (*n* = 67)
Time from injury to definitive fixation (days)	10.49 (1–45)	9.11 (1–43)	11.25 (1–45)
Time from injury to soft tissue cover (hours) ^a^	49.5 (14–120)	49.5 (14–120)	N/A
Time from injury to FRI diagnosis (days)	83.1 (12–475)	63.48 (12–145)	93.35 (32–475)
Time from FRI diagnosis to bone infection team review (days)	7.68 (0–25)	6.97 (0–18)	8.05 (0–25)
Acute infection (onset < 6 weeks)	30 (29.4%)	21 (60.0%)	9 (13.4%)
Chronic infection (onset > 6 weeks)	72 (70.6%)	14 (40.0%)	58 (86.6%)
Recurrent infection	21 (20.6%)	7 (20%)	14 (20.9%)
Implant retained			
Yes	49 (48.0%)	18 (51.4%)	31 (46.3%)
No	53 (52.0%)	17 (48.6%)	36 (53.7%)
Non-union requiring further surgery	10 (9.8%)	6 (17.1%)	4 (6.0%)
Signs of systemic sepsis	16 (15.7%)	5 (14.3%)	11 (16.4%)
Amputation	3 (2.9%)	1 (2.9%)	2 (3.0%)
Elevated ESR ^b^	63 (61.8%)	20 (57.1%)	43 (64.2%)
Elevated CRP ^b^	87 (85.3%)	30 (85.7%)	57 (85.1%)
Elevated WBC ^b^	80 (78.4%)	24 (68.6%)	56 (83.6%)

^a^ Open fractures only; ^b^ Normal ranges for ESR, CRP, and WBC are 1–14 mm/h, 0–6 mg/L, and 3.6–10.5 × 10^9^/L, respectively.

**Table 6:** Univariable and multivariable logistic regression analyses.

*n = 102*	FRI Treatment Failure					
	*Univariable*			*Multivariable*		
	Treatment Failure (*n* = 24)	Treatment Success (*n* = 78)	*p*-Value	Odds Ratio	95% CI	*p*-Value
Age at injury (years)	48.88	51.58	0.521			
Male gender	15 (54.2%)	51 (64.1%)	0.796			
BMI	31.88	25.86	0.186			
BMI ≥ 30	12 (50.0%)	20 (25.6%)	**0.025**	2.522	0.259–4.816	**0.006**
Smoker	11 (45.8%)	34 (43.6%)	0.847			
Diabetes Mellitus	9 (37.5%)	22 (28.2%)	0.408			
Open fracture	8 (33.3%)	27 (34.6%)	0.908			
Gustilo Anderson						
Type 2	0 (0%)	6 (7.7%)	**0.080**			
Type 3a	5 (20.8%)	8 (10.3%)	**0.091**			
Type 3b	5 (20.8%)	6 (7.7%)	**0.031**			
Type 3c	3 (12.5)	2 (2.6%)	**0.033**	4.683	2.037–9.784	**0.004**
Time to definitive fixation (days)	7.73	11.29	0.505			
External fixation as primary management	5 (20.8%)	10 (12.8%)	0.332			
Culture-negative	1 (4.2%)	4 (5.1%)	0.849			
Polymicrobial infection	14 (58.3%)	20 (25.6%)	**0.011**			
Implant retention ^a^	14 (77.8%)	35 (50.7%)	**0.048**	2.818	1.588–7.928	**0.041**
Polytrauma (ISS ≥ 16)	12 (50.0%)	37 (47.4%)	0.826			

^a^ Calculated for those who were treated by open reduction internal fixation (total *n* = 87; treatment failure *n* = 18; treatment success *n* = 69); FRI: fracture related infection; BMI: body mass index; ISS: injury severity score.

## Data Availability

The datasets used and analyzed during the current study are available from the corresponding author on reasonable request.
